# Structural heterogeneity of the Rieske iron–sulfur protein in a yeast complex III_2_

**DOI:** 10.1107/S2052252522012003

**Published:** 2023-01-01

**Authors:** Kutti R. Vinothkumar

**Affiliations:** a National Centre for Biological Sciences, Tata Institute of Fundamental Research, Bengaluru, India

**Keywords:** Rieske iron-sulfur proteins, yeast complex III_2_

## Abstract

Cryo-EM has been used to analyze the positions of the hydrophilic domain of the Rieske iron–sulfur protein in a yeast complex III_2_ under various redox and substrate/inhibitor conditions.

The enzyme ubiquinol:cytochrome *c* oxidoreductase, commonly called complex III is found in the inner membrane of mitochondria and bacteria, and is part of the electron transport chain. It accepts reduced lipophilic quinones from various de­hydrogenases. The enzyme catalyzes the transfer of an electron to cytochrome *c* from ubiquinol and, in this process, it also transfers protons across the gradient, which is used in the synthesis of ATP by ATP synthases. In plants, the complex cytochrome *b*_6_ *f* carries out the equivalent function (Sarewicz *et al.*, 2021 ▸). Complex III is a homodimeric protein with conserved core subunits of cyt *b* and cyt *c*_1_ and the Rieske iron–sulfur protein (ISP). ISP is a single-pass transmembrane protein, and the hydro­philic region of ISP contains the iron–sulfur (FES) cluster and is domain-swapped. As commonly observed in many complexes, the core subunits are surrounded by a number of supernumerary subunits in the eukaryotic complex III (Fig. 1 ▸).

At the heart of the complex are the low and high potential heme groups in cyt *b* and cyt *c*_1_ proteins, and the 2Fe–2S cluster in the ISP, which are responsible for electron transfer. The mechanism of how the electron transfer occurs and the proton translocation is explained by the ‘Q cycle’ proposed by Peter Mitchell (Mitchell, 1975 ▸; Crofts, 2021 ▸). There are two ubi­quinone binding sites in the cyt *bc*_1_ called Q_o_ and Q_i_. Ubiquinol binds to the Q_o_ site (quinone oxidation site) and transfers one electron to heme *c*_1_ via 2Fe–2S, subsequently reducing cytochrome *c*. Ubi­quinone at the Q_i_ site is reduced by the second electron via heme *b*_L_ and heme *b*_H_ generating a semi­quinone intermediate. This is accompanied by the translocation of two protons to the intermembrane space (IMS) from the Q_o_ site. The semi­quinone intermediate at the Q_i_ site is further reduced by the binding of a second ubiquinol to the Q_o_ site and uptake of protons from the mitochondrial matrix as well as two protons released from the Q_o_ site to IMS. During the enzymatic process, the ISP undergoes a large structural change to occupy multiple positions and the two extreme positions are called b and c to denote the proximity to the cyt *b* and *c*_1_. The necessity for such movement in intramolecular electron transfer has been supported by mutagenesis as well as with inhibitors (see reviews, Sarewicz *et al.*, 2021 ▸; Crofts, 2021 ▸; Kao & Hunte, 2022 ▸).

Complex III is one of the well studied membrane protein complexes by biochemical, biophysical and structural techniques (Sarewicz *et al.*, 2021 ▸). A number of crystal structures and recently cryoEM structures either in isolation or as part of supercomplexes from different organisms have been determined (reviewed in Kao & Hunte, 2022 ▸). The mobility of the ISP hydro­philic domain has been known from the early crystal structures but the fine details of the mechanisms at the Q_o_ site, the nature of proton translocation from and to the Q sites, and the cross-talk between monomers are areas where there are questions that remain to be answered (Sarewicz *et al.*, 2021 ▸).

In this issue of **IUCrJ** , Wieferig and Kühlbrandt undertake a comprehensive study of the *Yarrowia lipolytica* complex III_2_ by determining multiple structures with bound substrates/substrate mimics in different redox conditions by cryoEM and analyze the dynamics of the ISP (Wieferig & Kühlbrandt, 2023 ▸). A total of 9 samples of the enzyme were prepared including that in the apo state, with inhibitors and substrate/product bound, and under reducing (ascorbate) and oxidizing (ferrocyanide) conditions. The atovaquone (a Pf-type inhibitor that binds at the Q_o_ site) and antimycin A (a Q_i_ inhibitor) act as the reference structure, where the hydro­philic domains of ISP from both monomers are locked in one conformation. The consensus map from all the particles of different samples (except the atovaquone bound complex) yielded an overall resolution of ∼2 Å allowing the modelling of water molecules and lipids, and giving an atomistic view of complex III from this species of fungi [Figs. 1(*a*) and 1(*b*) ▸].

The cryoEM maps, as expected, revealed poor density for the ISP in many of these conditions, and using focused classification and signal subtraction during image processing, the populations of the enzyme with ISP in different positions in the complex could be separated. The maps of ISP in position b are of higher quality than those when it occupies the intermediate or c position, perhaps due to the interaction with the Q_o_ site and cyt *b*. The typical positions occupied by ISP in the enzyme are shown in Fig. 1(*c*) ▸, with 2Fe–2S cluster closer to the Q_o_ site in the b position and to heme *c*_1_ in the c position. Some observations include the lack of ISP in the b position in presence of decyl­ubiquinol and antimycin A treated enzyme, more prominent density for quinone at the Q_o_ site in reduced conditions (in the presence of ascorbate), ubi­quinone observed in the Q_o_ site and decyl­ubi­quinone in the Q_i_ site (quinones in both the sites are not modelled/observed in the same map). Structural analysis shows that the redox state of the enzyme and the substrate/inhibitor bound in the Q sites play a critical role in the interaction of ISP with cyt *b* and cyt *c*_1_. Further, the data also show that two ISP domains in the dimer are independent (at least in the isolated complex from this species), and both symmetric and asymmetric molecules are observed.

The preparation of defined samples and the possibility to analyze the different conformations/populations of the enzyme is one of the major advantages of cryoEM. Although the large movement of ISP has been well documented in earlier studies (Sarewicz *et al.*, 2021 ▸; Kao & Hunte, 2022 ▸), the extent of heterogeneity observed in the samples can be analyzed because of the ability to collect large data sets in a reasonable time frame due to detectors with faster frame rate and, more importantly, image processing algorithms. In future experiments, with careful sample preparation, *i.e.* not just with incubation but with faster mixing of substrate and enzyme followed by freezing (Crofts, 2021 ▸), it may be possible to observe substrate/product in both the Q sites and perhaps being able to follow the whole Q cycle can now be envisioned.

## Figures and Tables

**Figure 1:**
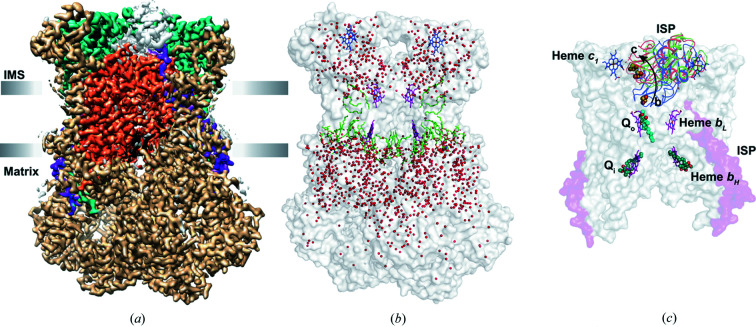
(*a*) CryoEM map of *Yarrowia lipolytica* complex III dimer. The consensus map (EMD-15312) of the complex at an overall resolution of 2 Å is shown. The map was calculated with *deepEMhancer* (Sanchez-Garcia *et al.*, 2021 ▸) from the two half-maps. The core subunits of complex III, cytochrome *b*, *c*_1_ and ISP are coloured orange, green and purple, respectively. The supernumerary subunits are coloured in tan and the non-protein densities are in black. Unmodelled densities are shown in grey, in particular the blurred density of the ISP hydro­philic domain can be seen. (*b*) The surface representation of the consensus model (PDB entry 8ab6) of complex III is shown in white. The co-factors and lipid/detergent molecules are shown in stick representation (the carbon atoms in heme *b*_L_ and heme *b*_H_ in purple, high potential heme *c*_1_ in blue, and lipid/detergent molecules in green). The water molecules are shown as red spheres. In the consensus model, only the transmembrane helix of the Rieske protein has been modelled and hence the 2Fe–2S cluster is not shown. (*c*) The surface representation of the core subunits of complex III with the TM helix of ISP coloured in purple. Typically observed positions of the hydro­philic domain of ISP are shown (from antimycin-treated samples) from b position to c position (blue, green and red for b, intermediate and c positions, respectively, are shown in cartoon representation). The 2Fe–2S cluster is shown as spheres and the movement of this cluster is shown with an arrow. The quinones in the Q_i_ site (green) and the Q_o_ site (cyan) are from different models (PDB entries 8abk and 8ab8, respectively) but shown in this figure to highlight the quinone binding sites. Figures were prepared with *Chimera* (Goddard *et al.*, 2007 ▸) and *PyMOL* (DeLano, 2002 ▸).
